# Chemosensory behaviour of juvenile crown-of-thorns sea star (*Acanthaster* sp.), attraction to algal and coral food and avoidance of adult conspecifics

**DOI:** 10.1098/rspb.2024.0623

**Published:** 2024-05-29

**Authors:** M. Webb, M. Clements, P. Selvakumaraswamy, E. McLaren, M. Byrne

**Affiliations:** ^1^ School of Life and Environmental Sciences, The University of Sydney, Sydney, New South Wales, Australia

**Keywords:** chemical ecology, asteroidea, COTS, olfaction, semiochemicals, choice chamber

## Abstract

Intraspecific and habitat-mediated responses to chemical cues play key roles in structuring populations of marine species. We investigated the behaviour of herbivorous-stage juvenile crown-of-thorns sea stars (COTS; *Acanthaster* sp.) in flow-through choice chambers to determine if chemical cues from their habitat influence movement and their transition to become coral predators. Juveniles at the diet transition stage were exposed to cues from their nursery habitat (coral rubble-crustose coralline algae (CCA)), live coral and adult COTS to determine if waterborne cues influence movement. In response to CCA and coral as sole cues, juveniles moved towards the cue source and when these cues were presented in combination, they exhibited a preference for coral. Juveniles moved away from adult COTS cues. Exposure to food cues (coral, CCA) in the presence of adult cues resulted in variable responses. Our results suggest a feedback mechanism whereby juvenile behaviour is mediated by adult chemical cues. Cues from the adult population may deter juveniles from the switch to corallivory. As outbreaks wane, juveniles released from competition may serve as a proximate source of outbreaks, supporting the juveniles-in-waiting hypothesis. The accumulation of juveniles within the reef infrastructure is an underappreciated potential source of COTS outbreaks that devastate coral reefs.

## 1. Introduction

Intraspecific, interspecific and habitat-mediated animal responses to chemical cues play key roles in aquatic environments in structuring populations and communities [1–4]. Chemical signals mediate species’ behaviour across planktonic and benthic life stages [1,5,6]. Larval settlement and juvenile development habitats are often quite different and separate from adult habitats, and there can be considerable spatial distances between them [7,8]. Targeted settlement of marine larvae is often promoted by chemical cues emanating from specific substrates and microhabitats that provide protection for early post-larvae and juveniles, as well as a food source for developing juveniles [1,9–13]. These nursery habitats are critical for populations of marine species as well as for their management and conservation [8]. Juvenile invertebrates may remain in the nursery habitat for some time delaying their growth as they subsist on local resources before migrating to the adult habitat when they switch to a new diet and grow to maturity [9,14–16]. For predatory sea stars, this prompted the ‘juveniles-in-waiting’ hypothesis. This hypothesis posits that juvenile populations can build up in nursery habitats in the reef infrastructure over recruitment years depending on prey availability and competition from adults [15,16]. While the sensory biology and chemical ecology of invertebrate larval settlement are well studied [1,6], the environmental and intraspecific cues underlying the switch from the juvenile nursery to the adult habitat are not well studied, although interference mediated by conspecific cues is implicated for several species [17–19].

The mechanisms underlying the transition from juvenile to adult habitat and the role of chemical cues are important to understand for ecologically influential species that in aggregations can overgraze algae or decimate prey species leading to economic losses (e.g. bivalve fisheries) and ecosystem change [20–24]. As shown in many behavioural studies, marine species often respond to chemical cues originating from a distance without direct contact with the source, and so information pathways can be difficult to unravel [3,25]. Understanding species’ responses to environmental olfactory information has applications in fisheries and aquaculture and is used in management to disrupt nuisance species [3,26] with interest in application to pest sea stars [27–29].

The chemical ecology of sea stars as predators or as prey is well investigated. Studies have shown chemically mediated attraction to prey, fleeing or alarm responses away from predators and conspecifics, and attraction between mature individuals that promotes aggregation and synchronized spawning [25,28,30–34]. For the crown-of-thorns sea star (COTS; *Acanthaster* sp.), foraging behaviour is mediated by chemoattractants released from coral prey and by conspecifics preying on coral [35,36], while reproductive behaviour is mediated by pheromones [32,37]. As outbreaks of *Acanthaster* sp. drive a major decline in coral cover and shifts in community structure and ecosystem function [22], the behaviour and sensory biology of this species have been investigated with most studies on the adult stage [31,35,36]. The population dynamics of COTS are likely to be mediated by intraspecific semiochemical cues, where signals released by individuals convey information that elicits a behavioural change in conspecifics [3,28]. There is considerable interest in the development of targeted mitigation strategies, beyond current adult culling approaches, to address the environmental and economic impacts of COTS, including the potential use of semiochemicals [28,29].

Research into the cause(s) of COTS outbreaks has largely focussed on the larval and adult stages [24,38,39]. The potential that increased larval food driven by nutrient run-off enhances that their success has prompted efforts to improve water quality, although, the link between run-off events and COTS outbreaks is equivocal [40–42]. For the adult stage, overfishing of predators as a cause of outbreaks has gained traction through tracing COTS eDNA in predatory fishes [43]. Recent research points to the potential role of reservoirs of juvenile recruits in driving outbreaks [15,44].

*Acanthaster* start their benthic life as algae eaters with a preference for crustose coralline algae (CCA) [15,45–47]. In the absence of coral prey, they can remain as herbivores for years (at least 6.5 years), delaying their ontogenetic switch to corallivory [15,47]. There is potential for cohorts of juveniles to accumulate as a ‘hidden army’ in the reef infrastructure (juveniles-in-waiting). This phenomenon occurs in other predatory sea stars (*Asterias, Marthasterias*) where juveniles can remain in the recruitment-nursery habitat for months to years at growth stasis before joining the adult population [14,16,44]. This delay is suggested to indicate a negative density-dependent feedback control of population dynamics that may involve adult–juvenile semiochemical signals [16,44]. The inability to target COTS across its benthic phases in mitigation programmes emphasizes the need for increased knowledge of the juvenile stage to improve and/or augment current control methods [28].

We investigated the behaviour of juvenile herbivorous-stage COTS in response to a range of waterborne chemical cues that they had not previously experienced to determine if innate behaviour may be involved in habitat ‘recognition’. These experiments involved juveniles at the size/age for transition to corallivory [15]. It is important to understand what promotes herbivorous juveniles to become coral predators, and we investigated the potential that chemical ecology is involved. Using flow-through choice chamber experiments, we provided the juveniles with cues from coral rubble covered with CCA (nursery habitat), live coral and adult COTS in various combinations to determine their ability to respond to waterborne chemical stimuli. We assessed if these cues can be detected by the juveniles and influence their ‘decision’ with respect to movement, direction and locomotory speed towards or away from the source of the cue. Juvenile COTS can detect the presence of algae and live coral [45,48] and so we expected that they would be attracted to olfactory information from these sources delivered inflow from a distance. Studies of adult COTS (>15 cm diameter) show that intraspecific chemical cues influence their behaviour (review in [28]), and so we also expected that the juveniles would detect and respond to cues emanating from adults.

This is, to our knowledge, the first study to explore the potential for conspecific interactions between juvenile and adult sea stars and is one of the few that have investigated the behavioural interactions between these life stages for a marine invertebrate [49,50]. We aimed to determine if innate behavioural responses mediated by chemosensation mediate life-stage diet and habitat transitions in juvenile COTS. On the one hand, we expected that the juveniles would be attracted to the adults as a signpost to the presence of coral prey, and on the other hand, that they may be repelled by the adults to avoid competition, indicating density-dependent feedback in migration to the adult habitat and diet.

## 2. Material and methods

### (a) Specimens and rearing conditions

As the taxonomy of the Pacific species of *Acanthaster* is uncertain [51,39] the species investigated here is referred to as *Acanthaster* sp. or COTS. Adults were collected near Cairns (northern Great Barrier Reef (GBR)) by COTS control divers and shipped to the Sydney Institute of Marine Science where they were maintained inflow through aquaria (filtered sea water (FSW) 5-µm) at 26°C, the temperature of their habitat (http://data.aims.gov.au/aimsrtds/yearlytrends.xhtml). For each of the three fertilizations, the gametes of two different males and two females were used, and the larvae were reared to the settled juvenile stage following routine methods [15]. Larvae with a well-developed juvenile rudiment (14–16 days post-fertilization) were induced to settle using coralline algae (*Amphiroa* sp.). The juveniles were maintained in a constant temperature room (26°C; 12 : 12 h light cycle) in UV sterilized 1 µm FSW with ambient salinity of approximately 34‰ (Merck salinity probe) and fed small fronds of *Amphiroa* sp. Juveniles, up to 3 mm diameter, were placed in 12-well dishes (5 ml wells). Food and water were renewed every 3 days. As the juveniles grew (>3 mm diameter), they were transferred to 6-well dishes (10 ml well), and when they reached 10 mm diameter, they were transferred to large culture dishes (100–200 ml). For the large juveniles, culture dishes, food and FSW were renewed twice a week.

### (b) Behaviour experiments

The behaviour experiments were conducted using a suite of cues including live coral, coral rubble encrusted with CCA and adult COTS. The CCA-covered rubble was collected from the shallow reefs (<2 m depth) at One Tree Island, GBR (23.5076° S, 152.0916° E) and maintained in a tropical aquarium at 26°C. Corals (*Acropora* sp.) that are the preferred prey of COTS [52] were sourced from aquarium suppliers. The CCA and live corals were maintained in separate 20 l closed system aquaria at 26°C. Adult COTS were maintained in a 160 l tropical aquarium system at 26°C. Prior to the start of the coral and CCA rubble experiments, these cues were maintained for 2 h in the header tank (25 l) source water before turning on the flow. The amount of rubble and live coral used covered the base of the header tank as similarly as possible between trials to emulate a layer of habitat in nature. Adult COTS were placed in the header source for 30 min prior to the start of the experiment. During this time, water circulation was maintained using a water pump.

The behavioural responses of the juveniles were tested in a constant temperature room (26°C) using a two-channel choice flume as used in behavioural studies of fish larvae and sea urchin juveniles [5,49] (details in the electronic supplementary material, S1). Each flume was supplied with a steady gravity-driven flow (8.3 cm^3^ s^−1^ per channel) controlled by a flow meter (Dwyer MMA series) at the FSW input. Laminar flow was established at the inflow point by a course filter pad lined with a mesh screen. The separation of the two streams was confirmed with dye. To ensure that behaviour was not influenced by vision [53], the experiments were done under red light 625 λ (measured by Ocean Optics, Spectrophotometer USB4000; Ocean View 2.0 software), which exceeds the photoreception range of COTS eyes [31]. Prior to use in trials, the juveniles were placed in clean containers of 1.0 µm UV FSW with no food for 24 h. These are also the conditions they experienced when first placed in the choice flume prior to the turn on of flow. All experiments were conducted during the day.

The water flow on each side of the flume was segregated by a divider which ensured parallel flow into the main chamber supporting a distinct distribution between the cue source flows and eliminated backflow (electronic supplementary material, file S1). Each side of the flume was supplied with water from a 25 l header tank, that contained a treatment cue. At the start of each experimental run, a single juvenile was placed in the centre of the main chamber where it would receive even flow from both sides of the flume. At the point of water outflow, a gate was installed to allow the juvenile to acclimate for 1 min in static water. The gate was then removed, and the flow was turned on. Although the exact position of the juveniles when the gates were lifted, varied somewhat, we did not adjust to avoid physical disturbance. None of the juveniles were in a location where they would only experience flow from one side of the flume.

Juveniles were allocated 20 min to respond as demonstrated by upstream movement towards the cue source or downstream movement. The treatment cues; live coral, CCA reef rubble, and COTS, were tested individually against FSW only. Treatment cues were then tested against each other (CCA/live coral; CCA/COTS and COTS/live coral). The same juvenile was not used more than once per treatment. Treatment cues were rotated between the left and right sides of the flume to remove a side bias. Images taken at 20 s intervals after the acclimation period were captured with an Olympus Tough TG-6 (Olympus) camera and were then autogenerated into time-lapse videos. The camera was mounted above the choice flume with the entire arena and scale in view. Three juveniles climbed the chamber wall and ‘surfed’ at the air–water interface oral side up and so were not used for the experiment.

Juvenile size (diameter mm) was similar but the number of replicates varied somewhat among treatments (FSW *$\bar{x}$* = 10.32, s.e. = 0.40, *n* = 42; COTS $\bar{x}$ = 10.24, s.e. = 0.42, *n* = 50; coral *$\bar{x}$* = 9.67, s.e. = 0.21, *n* = 30; coral/COTS *$\bar{x}$* = 9.67, s.e. = 0.21, *n* = 41; CCA *$\bar{x}$* = 9.40, s.e. = 0.19, *n* = 27; CCA/coral $\bar{x}$ = 12.97, s.e. = 0.25, *n* = 35; CCA/COTS *$\bar{x}$* = 12.77, s.e. = 0.36, *n* = 22). We conducted more runs for the adult COTS cue to facilitate the interpretation of juvenile responses because this treatment was the most variable.

Juvenile movement in the time-lapse files was analysed using Tracker (v. 6.0.1). Each file was calibrated in Tracker to display at a framerate of 1.5 s^−1^. Path length (m) was manually calculated from the starting point until the juvenile moved towards or away from the direction of flow. Tracking was used for single and two-cue treatments for the juveniles that remained in the field of view for the entire time and so could be completely followed (*n*: coral = 11, CCA = 14, COTS = 20, FSW = 8, CCA/coral = 13, CCA/COTS = 12 and coral/COTS = 12). The paths taken by the juveniles were traced and illustrated (electronic supplementary material, S2) as in a previous study of sea star behaviour [54].

### (c) Statistical analyses

All analyses were performed using R (v. 1.2.1335) [55]. A chi-squared goodness-of-fit test was used to analyse the percentage frequency of the juvenile choice responses with equal expected proportions. Choice response data were analysed with 25%:25%:25%:25% probability of upstream movement to either treatment cue, downstream or no movement. For the FSW only (control), the data were analysed with 33%:33%:33% probability of upstream movement, downstream movement or no movement. The choice data were illustrated as the percentage of individuals in each response (e.g. to cue, downstream, no movement).

Tracking data on speed (mm min^−1^), distance moved (mm) and time to make a choice (min) were analysed. For the single-cue experiments, only the significant responses (to or away from cue) as indicated by chi-squared analysis were tracked. The speed data were analysed by a one-way analysis of variance (ANOVA) with treatment as a fixed factor with four levels (attraction to coral, attraction to CCA, upstream movement in FSW only, avoidance to adult COTS) with the FSW as the control. Homogeneity of variance (HOV) and normality were confirmed by Levene’s and Shapiro–Wilk tests, respectively (significance *α* = *p* < 0.05). Tukey’s honestly significant difference(HSD) post hoc-pairwise comparison were performed to identify treatments that differed. Data on distance moved and time to make a choice did not meet the assumption of HOV, and so these data were analysed using Kruskal–Wallis non-parametric ANOVA with treatment type as a fixed factor. Dunn’s test (R package FSA) with Bonferroni-corrected *p*-values was used post hoc to identify significant pairwise treatment effects.

For the two-cue experiments, data on distance moved, time to make a choice and speed were analysed by ANOVA with treatment as a fixed factor with three levels (CCA/live coral, CCA/COTS, live coral/COTS). Assumptions of HOV and normality were confirmed as above, except for the normality of the distance moved data. As ANOVA is robust to this [56], the analysis was undertaken, and a Tukey HSD post hoc-pairwise comparison was performed.

## 3. Results

### (a) Choice response

**Figure 1.**
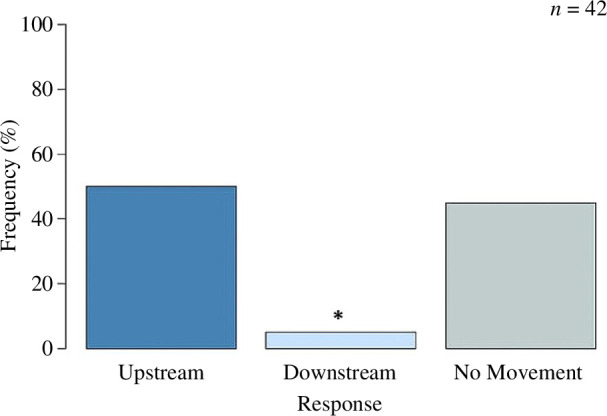
The percentage frequency (%) of COTS juvenile responses when exposed to FSW only, analysed against an expected distribution (equal probability of 33%) of upstream, downstream and no movement (chi-squared goodness-of-fit test, ‘asterisk’ indicates significance).

In the FSW-only treatments, the juveniles that did move (55%, *n* = 42) showed a preference for upstream movement with 21 individuals moving upstream and only two moving downstream. A significant number of juveniles did not move (*χ* ^2^ = 15.57, *p* = <0.05; figure 1).

**Figure 2.**
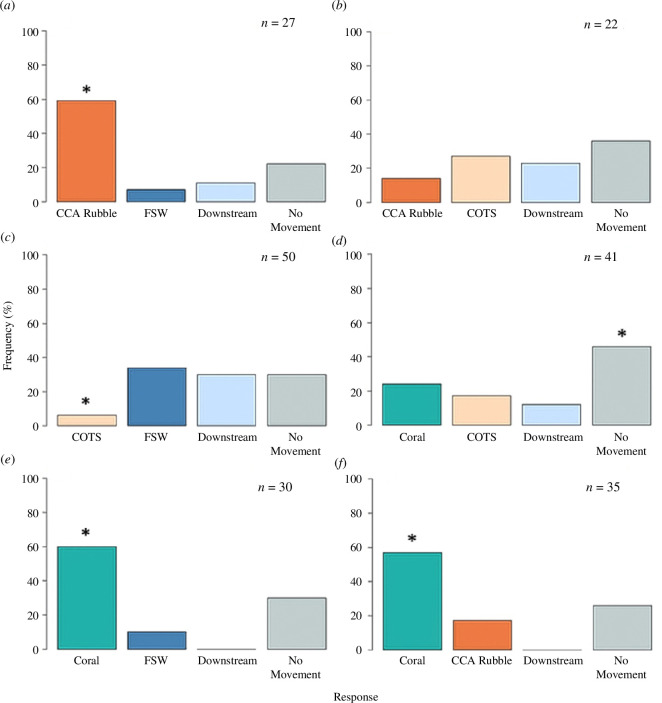
The percentage frequency (%) of COTS juvenile choice response analysed against an expected distribution (equal probability of 25%) of movement to cue, upstream (FSW), downstream, or no movement (chi-squared goodness-of-fit test, ‘asterisk’ indicates significance). (*a*) In response to CCA rubble as a single cue, most juveniles moved to CCA rubble. (*b*) When two cues are present, CCA rubble and COTS, there was no significant choice. (*c*) In response to adult COTS as a single cue, most juveniles appeared to avoid this cue. (*d*) When two cues are present, coral and adult COTS, most juveniles did not move. (*e*) In response to coral as a single cue, most juveniles moved to coral. (*f*) When two cues are present, coral and CCA rubble, most juveniles move to coral.

In the single-cue experiments (versus FSW), the juveniles demonstrated significant attraction to live coral and CCA-coral rubble (*χ* ^2^ = 25.2, *p* = <0.005 and *χ* ^2^ = 18.2, *p* = <0.005, respectively) and avoidance to cues from adult COTS (*χ* ^2^ = 9.84, *p* = <0.05; figure 2*a*,*e*). For CCA rubble (*n* = 27) and coral (*n* = 30) presented as single cues, approximately 60% of the juveniles moved towards these cues with movement downstream or into the FSW flow being far less prevalent. In these treatments, 22 and 30% of juveniles remained stationary, respectively. Of the 50 juveniles that were exposed to cues from adult COTS, 34% moved away from the cue upstream to FSW, 30% moved downstream, 30% did not move and a significantly lower number (6%) moved towards the adult cue (figure 2*c*).

In the CCA rubble-live coral two-cue experiment, the choice response for coral was threefold greater than that for CCA rubble (*χ* ^2^ = 24.1, *p* = <0.005; figure 2*f*). Overall, 57% (*n* = 35) of the juveniles moved towards coral, similar to that for the coral-only runs, and 26% of juveniles remained stationary. In the coral/COTS trails, the no movement (46%) category was significant (*χ* ^2^ = 11.2, *p* = <0.05). In the two-cue experiments where one of the cues was adult COTS, direct observation indicated that the juveniles had a meandering movement, and their responses were variable (figure 2*c*). When simultaneously presented with adult COTS and the CCA rubble cue, there was no significant choice (figure 2*b*). Across all combinations of cues, a similar percentage ( $\bar{x}$ = 34%, s.e. = 0.35, *n* = 7 trial types) of juveniles remained stationary with their arm tips raised sensing the environment.

### (b) Choice movement analysis

**Figure 3.**
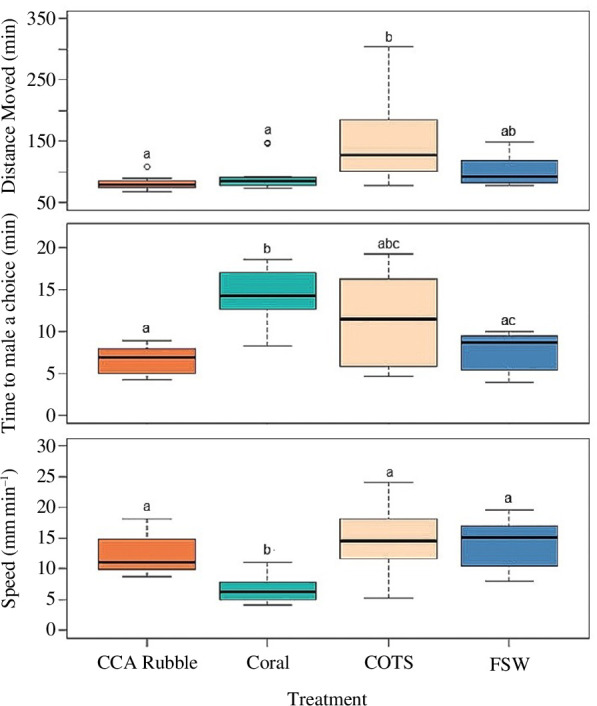
Data on (*a*) total distance moved (mm), (*b*) time to make a choice (min), and (*c*) speed (mm min^−1^) of juvenile COTS to single treatments: CCA-covered coral rubble (*n* = 14), live coral (*n* = 11), adult COTS (*n* = 20) and FSW (*n* = 8). Letters indicate treatments that differed significantly (Tukey’s HSD test (speed); Kruskal–Wallis/Dunn’s test (distance, time)).

In the single-cue experiment, there was a significant effect of cue type on juvenile movement (figure 3). The pathways that the juveniles took are illustrated in the electronic supplementary material, S2.

There was a significant difference in the speed of juveniles in the single-cue experiments with the movement to the coral cue being the slowest ( $\bar{x}$ = 6.8 mm min^−1^, s.e. = 0.7, *n* =11; ANOVA, *F*_3,49_ = 9.68, *p* = <0.01) while the other treatments did not differ (figure 3*c*). In the FSW controls and CCA-coral rubble treatments, the juveniles moved upstream at an average rate of 14.2 mm min^−1^ (s.e. = 1.5, *n* = 8) and 12.6 mm min^−1^ (s.e. = 0.8, *n* =14), respectively. While it is difficult to separate the contributions of the physical stimulus from flow and olfactory information in the upstream movement towards CCA-coral rubble and live coral, the dominant movement was into the arm of the flume from which the flow plus cue was delivered (electronic supplementary material, S2). The juveniles moved away (downstream and into FSW flow) from the adult COTS cue source at an average rate of 14.5 mm min^−1^ (s.e. = 1.1, *n* = 20).

The time to make a choice was also influenced by the treatment cue (Kruskal–Wallis *χ* ^2^ = 17.94, *p* = <0.01) with the time to move towards the coral cue being the slowest ( $\bar{x}$ = 14.5 min, s.e. = 0.9, *n* = 11), while the other treatments did not differ (figure 3*b*). Time to make a choice was the fastest towards the CCA-coral rubble ( $\bar{x}$ = 6.8 min, s.e. = 0.4, *n* = 14). The slowest response recorded was for juveniles moving away from the adult COTS cue ( $\bar{x}$ = 11.3 min, s.e. = 1.2, *n* = 20), but note that this was also the most variable (figure 3*b*).

The total distance moved by the juveniles also differed in the single-cue experiments (Kruskal–Wallis *χ* ^2^ = 24.35, *p* = <0.01) with the shortest and most direct paths taken were in response to the CCA-coral rubble, live coral and FSW treatments ( $\bar{x}$ = 80.9 mm, s.e. = 2.7, *n* = 14; *$\bar{x}$* = 94 mm, s.e. = 8.11, *n* = 11; and *$\bar{x}$* = 101.9 mm, s.e. = 8.7, *n* = 8; respectively), which did not differ. The total distance moved by the juveniles in response to the adult COTS was significantly longer ( $\bar{x}$ = 144.7 mm, s.e. = 12.9, *n* =20; figure 3*a*) owing to the meandering path they took moving away (downstream or upstream into the FSW-only flow) from the cue.

**Figure 4.**
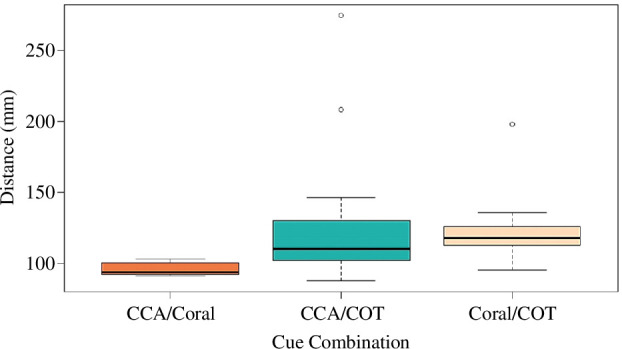
Total distance moved (mm) by the juveniles in response to two-cue combinations, CCA-covered coral rubble/live coral (*n* = 13), CCA/adult COT (*n* = 12) and live coral/COT (*n* = 12).

Similarly, in the two-cue experiments, the distance moved by juveniles was greater in the presence of the adult COTS cue (together with CCA-coral rubble or live coral) owing to their meandering movement. This contrasted with the shorter more direct movement in the CCA-coral rubble/live coral cue combination (ANOVA, *F*_2,34_ = 3.529, *p* = <0.05). In the CCA-coral rubble/COTS and live coral/COTS experiments, the mean distance moved was 131.0 mm (s.e. = 15.9, *n* = 12) and 124.1 mm (s.e. = 7.3, *n* = 12), respectively, while the distance moved in response to CCA-coral rubble/live coral was $\bar{x}$ = 96.4 mm (s.e. = 1.4, *n* = 13; figure 4). There was no significant difference in the time to make a choice (ANOVA, *F*_2,34_ = 0.6, *p* = 0.554) or speed (ANOVA, *F*_2,34_ = 0.48, *p* = 0.623) in the two-cue experiments.

## 4. Discussion

Waterborne cues convey environmental information to aquatic organisms, thereby eliciting behavioural change with semiochemicals being important in intraspecific communication [3,6,25,28,32,37,49]. For sea stars and other marine invertebrates, this is the best known for chemical cues that mediate behaviour of conspecific adults [28,57]. Far less is known about the role of chemical cues in olfactory communication between the adult and juvenile life stages which is important in the context of spatially segregated juvenile and adult habitats [8]. To help tease out potential responses to reef chemistry, we presented herbivorous-stage juvenile COTS with olfactory cues in various combinations. We show that they have distinct chemosensory capabilities being able to respond to novel olfactory cues not previously encountered in their life delivered from a distance through a flowing current. Our findings indicate the presence of innate behaviour in the juveniles in response to a range of cues.

As typical of sea stars [25,54,58], juvenile COTS exhibit positive rheotaxis to water flow. They also responded to cues that signpost the location of their food/nursery area (CCA-covered coral rubble), the presence of coral prey and adults. In response to rubble-CCA and coral as single cues, the juveniles moved towards the source of these cues and, when these two cues were presented in combination, they exhibited a preference for coral. As nursery habitats are important for juvenile survival [8] and the switch to coral prey is an essential step in transitioning to maturity in COTS, these responses were expected. By contrast, there was no clear indication of a ‘decision’ among juveniles that moved in the two-cue CCA-rubble/COTS and live coral/COT trials. The variable responses observed in these trials may reflect the situation in nature where the juveniles need to integrate multiple cues together with semiochemicals from adults. Interestingly, across all cue combinations, a similar percentage of juveniles remained stationary with their arm tips raised sensing the environment, as noted in a previous sea star study [54]. This may have been a response to the new environment (chamber) and may also indicate that, in a given situation, a proportion of juvenile COTS have a cautious immobile response potentially as a defence against being detected by predators and to take time to sense the environment.

Reef rubble covered by CCA attracts larval settlement in COTS and many other species [59], attributed to specific chemistry [1]. Settlement of the larvae to CCA places developing juveniles in their nursery habitat which provides them with food and shelter [13,47]. Juvenile attraction to the reef rubble cue was very strong despite months of rearing in isolation. The juveniles had the shortest path length and the fastest time to make a positive choice in response to the reef rubble cue. The source of this cue, rubble collected from the reef probably reflects the habitat of the juveniles in nature (see [47]). In addition to CCA, this habitat supports a diversity of encrusting biota, meiofauna, algal biofilm and microbes, to name a few sources of habitat chemistry. The attraction of the juveniles towards coral rubble is probably owing to the detection of a chemical mixture that conveys information as to the location of the nursery habitat.

The response of the juveniles to first exposure to cues from coral indicates an innate attraction to coral prey, as indicated in other studies [48,60]. While there was a definite attraction towards live coral (shortest distance moved), the time that the juveniles took to make this choice was the longest of any treatment and with the slowest average speed, indicating cautious attraction. This cautionary approach is warranted. The transition to a coral diet is perilous for juvenile COTS as they can be damaged or killed by coral defences (nematocysts, mesenterial filaments and sweeper tentacles) [48,60–63]. Some corals can inflict 100% mortality of juvenile COTS [48]. Injured juveniles may revert to an algal diet while they heal and regenerate, a process that can take weeks [48,60]. Even the preferred *Acropora* prey of COTS can inflict serious damage to juveniles [60]. For those juveniles that survive, healing the damage inflicted by coral is another factor in the delay to becoming coral predators.

We were uncertain as to how the juveniles would respond to adult chemistry, attraction or avoidance. When exposed to olfactory information from adults as a single-cue source, 94% of the juveniles moved downstream away from the source of the cue, into the no-cue FSW flow or remained still. These results indicate the avoidance of adult conspecifics. The tendency to move upstream seemed to be overridden in some juveniles as they moved downstream when adult chemistry was in the flow stream. This avoidance may indicate that juvenile COTS face the risk of being preyed on by adults as cannibalism is common in predatory sea stars [34,64]. The reasons why juveniles appeared to flee from adult cues are unknown but our results indicate that olfactory information from adults, at least in isolation or close proximity may impede the ontogenetic transition to corallivory. These results contrast with the mutual attraction between adult COTS where individuals move towards feeding or spawning conspecifics, an attraction promoted by semiochemicals which can result in dense aggregations [31,32,35,37]. Our results also contrast with that seen for sea urchins where juveniles are attracted to and shelter under adults [49].

We were most interested as to how the juveniles would respond to cues from live coral together with adult cues, given that they were at the diet transition stage, and their strong positive and negative responses to these cues as individual stimuli, respectively. In the coral/adult COTS experiments, the juveniles exhibited variable responses. While more juveniles moved towards the coral than the adult cue, downstream movement also occurred, and no movement was significant. There may be interindividual variability in juvenile behaviour at the diet transition stage modulated by chemical cues across life stages (juveniles and adults) that in nature probably involve a plethora of olfactory information.

We have a poor understanding of the spatial distribution of juvenile COTS in nature and the conditions that they experience because they are very difficult to find. Despite extensive searches including destructive sampling efforts, herbivorous juvenile COTS are rarely seen [65,66], although a mass recruitment of juveniles was observed in Fiji [67] and a recent study on the GBR located juveniles on rubble in forereef habitat [63]. When juvenile COTS achieve competence to transition to a coral diet, they may have to navigate to find corals at a distance. In a tracking study, juveniles (15–40 mm diameter) migrated 800 m from their back reef nursery habitat towards the reef crest over approximately 2 years [67]. This migration was probably driven by waterborne cues from coral on the reef crest.

On the reef, juvenile COTS experience a complex suite of cues, and as radial animals, can receive olfactory information from many directions [54,58]. It would be of interest to investigate the responses of the juveniles to the predatory crabs that occur with them in the rubble [68]. Multiple simultaneous interactive cues are likely to influence behavioural decision-making that may differ between juveniles as seen here. These interactions and behavioural responses to multiple co-occurring cues warrant further investigation, including the potential for double-negative- or double-positive-cue feedback (see [69]).

Juvenile growth on an algal diet ceases at an asymptotic small size (<20 mm diameter) with algae-supporting maintenance only [15,45]. The juveniles are resilient to food scarcity, being able to delay their transition to corallivory until favourable conditions arise with no influence of a years-long algal diet on their growth once coral prey is available [15]. Juvenile transition to corallivory is likely to be influenced by the availability of coral prey, which would be unpredictable following COTS outbreaks, cyclones and bleaching events [22,70]. The collapse of the reef to coral rubble following bleaching mortality is followed by the overgrowth of algae [71]. Thus, herbivorous juvenile COTS living in the reef infrastructure may benefit from climate-change-driven coral mortality and increase in their nursery habitat and food [44]. Following recent mass-bleaching events on the GBR, *Acropora* species were the first corals to recover [72]. Importantly, this is also COTS-favoured prey [73]. Waiting-stage juveniles persisting in the rubble habitat post-bleaching would be well positioned to transition to corallivory in parallel with coral growth, impeding reef recovery. The appearance of new coral-eating juveniles (as small as 3 cm diameter) after a COTS outbreak has passed [74] is probably owing to the emergence of juveniles that were in the reef infrastructure. Our study provides insight into juvenile behaviour that may underlie these observations. The size range of COTS in outbreaks indicates the presence of multiple cohorts [75] and this is probably associated with the waiting stage phenomenon [15]. The behavioural ecology of juvenile COTS at the transition to corallivory is important in understanding and predicting outbreaks. An important question is what drives herbivorous juvenile COTS to become coral predators. Our results indicate that their sensory abilities are key. The potential accumulation of juveniles within the reef infrastructure over multiple spawning years (depending on survival) [15] is an underappreciated driver of COTS outbreaks.

For marine species, juvenile survival is crucial as this stage is considered to be a mortality bottleneck in population dynamics [8,76]. While juvenile COTS experience pressure from predation and disease [61,67,68], they are tolerant of other environmental stressors including heatwaves [44]. The propensity to outbreak followed by a population decline is a natural phenomenon for COTS [21,77]. In low densities, COTS may benefit coral diversity (e.g. intermediate disturbance hypothesis) [78]. However, outbreaks are a major driver of coral mortality and appear to have become more frequent [79,80]. With the inability to address coral demise owing to ocean heating (mass-bleaching mortality), controlling COTS through culling is one of the few management intervention tools available to help protect reefs in a tenuous state owing to climate change [27,81].

This study, together with the characterization of the biochemistry of adult COTS [31,82], contributes to knowledge to develop strategies as to how semiochemicals may be used as a management tool for COTS. For instance, chemical agents isolated from adults might be used to reduce the number of juveniles making the transition to becoming coral predators. While there are great complexity and ethical considerations with respect to use of biocontrol in the marine environment, the ability to manipulate population dynamics through the use of influential chemical agents has been used as a management tool for nuisance aquatic species [3,26].

As characteristic of marine species, density-dependent processes across life stages (settler, juvenile and adult density) and life stage interactions (e.g. competition, mortality and emigration) [8,19] undoubtedly influence COTS population dynamics. Conspecific interference competition can be a selective force in the migration of juvenile marine invertebrates from their nursery habitat [19,50]. Juvenile avoidance of adult cues indicates the presence of negative density-dependent feedback, whereby the presence of adult COTS deters recruitment into the adult habitat and diet. Juveniles may delay the transition to corallivory to avoid competition for food, especially if the adult density is high. Density-dependent ontogenetic transitions have been observed for other predatory sea stars in nature [14,16]. Importantly, our results indicate that the removal of adult COTS may release juveniles residing in the reef infrastructure from competition interference, thereby promoting their transition to corallivory. This scenario may contribute to an explanation as to why some reefs require multiple voyages by culling teams to clear COTS, especially small individuals [27]. Our findings lead to support the juvenile-in-waiting hypothesis [15]. Chemical ecology and the potential for cross-life stage communication may underlie the periodic outbreaks of COTS. Density-dependent feedback is likely to be key to the outbreak phenomenon.

## Data Availability

Data files that support this study are publicly accessible on Dryad [83]. Electronic supplementary material is available online [84].
